# Asymptomatic Cholecystocolonic Fistula: A Diagnostic and Therapeutic Dilemma

**DOI:** 10.1155/2013/754354

**Published:** 2013-04-17

**Authors:** Nicola Antonacci, Giovanni Taffurelli, Riccardo Casadei, Claudio Ricci, Francesco Monari, Francesco Minni

**Affiliations:** Department of General and Emergency Surgery, University of Bologna, S. Orsola-Malpighi Hospital, Via Massarenti 9, 40138 Bologna, Italy

## Abstract

Cholecystocolonic fistulas (CCF) are rare complications of gallstones with a variable clinical presentation. Despite modern diagnostic tools, cholecystocolonic fistulas are often asymptomatic and it is difficult to diagnose them preoperatively. Biliary-enteric fistulae have been found in 0.9% of patients undergoing biliary tract surgery. The most common site of communication of the fistula is the cholecystoduodenal (70%), followed by the cholecystocolic (10–20%), and the least common is the cholecystogastric fistula. Herein, we report a case of female patient with multiple episodes of acute recurrent cholangitis due to common bile duct and gallbladder stones in which preoperative imaging studies were negative for cholecystocolonic fistula that was incidentally discovered and treated during surgery and was appropriately treated. A review of the literature is reported too.

## 1. Introduction

Cholecystocolonic fistula is a late complication of gallstone disease and is found in 1/1000 cholecystectomies. The incidental finding of cholecystocolonic fistula during cholecystectomy is rarely reported, ranging from 0.06% to 0.14% [1–3]. Nevertheless, CCF is the second most common cholecystoenteric fistula after the cholecystoduodenal [1–3].

## 2. Case Report

A 55-year-old female with history of gallstones came to the emergency room with diffuse right-upper abdominal pain without fever. On physical examination, her vital signs were stable, and she was afebrile. She was morbidly obese (BMI = 36) and had a nondistended abdomen. Blood tests were all within normal values except for an ALT of 400 (normal value <31 U/L) and an AST of 139 (normal value <32 U/L) and an increase serum gamma-GT (116 U/L; normal value 5–36 U/L) and direct bilirubin (3.44 mg/dL; normal value 0.00–0.30 mg/dL). Abdominal ultrasonography revealed multiple shadowing gallstones with a dilated common bile duct without intraluminal gallbladder air and pericholecystic fluid. 

**Figure 1:**
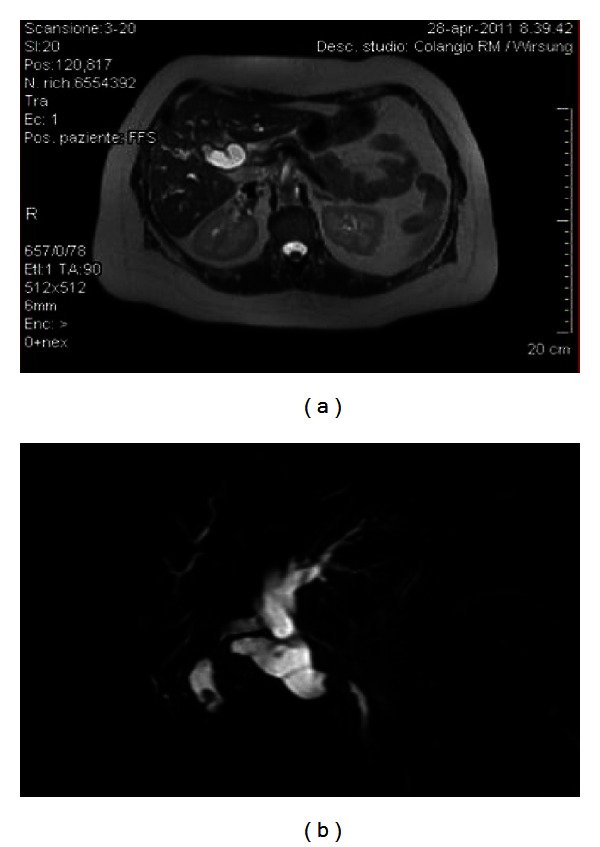
(a) MRI showing a sclero-atrophic cholecystitis with an endoluminal stone. (b) Cholangiographic reconstruction showed an absence of signal in the pre-papillary tract of CBD, with intrahepatic duct dilatation of the left lobe of the liver.

For the presence of a dilated common bile duct, her workup included a magnetic resonance (MRI) that showed common bile duct (CBD) lithiasis in the prepapillary tract of the common bile duct, 4 cm above the papilla of Vater associated with intrahepatic duct dilatation of the left lobe of the liver (Figures 1(a) and 1(b)). 

Subsequently, the patient underwent an endoscopic retrograde cholangiopancreatography (ERCP) to treat the CBD lithiasis through sphincterotomy and stone extraction. 

**Figure 2:**
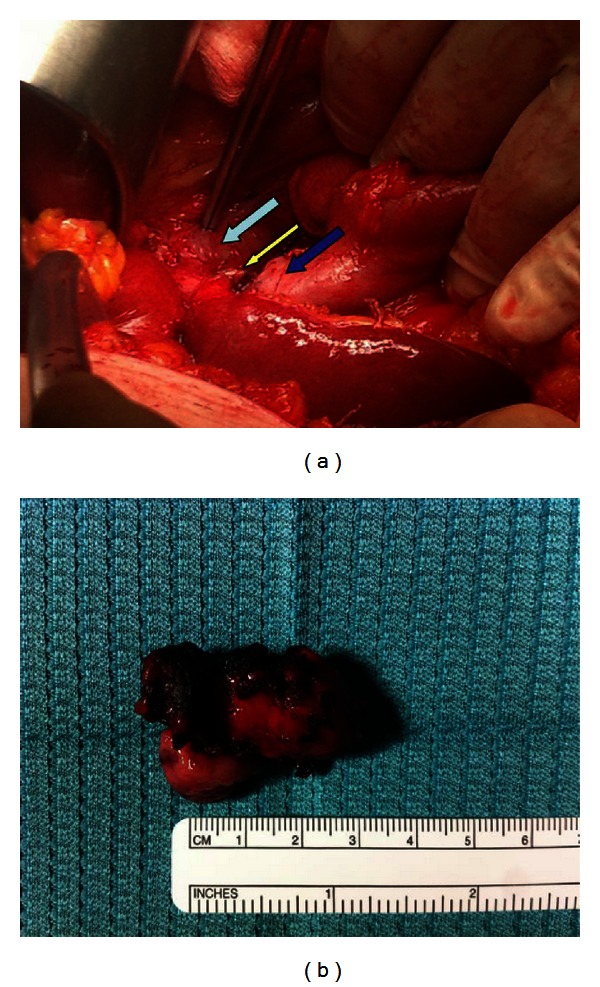
(a) Transverse colonic loop (blue arrow) tightly adherent to cystic duct (yellow arrow) gallbladder (light blue arrow). (b) Macroscopic appearance of the removal cholecystocolonic fistula.

After this procedure, the patient's clinical and laboratoristic aspects became normal. The patient underwent laparoscopic cholecystectomy, but, during surgery, a cholecystocolonic fistula was suspected because of a close connection between the gallbladder and the transverse colon. Thus, a laparotomy was performed, and the cholecystocolonic fistula was detected (Figure 2(a)) and treated with cholecystectomy and the resection of the colonic fistula with TA 45 stapler (Figure 2(b)). Postoperative course was uneventful, and the patient was discharged without complications on postoperative day 6. The pathological examination of the specimen showed chronic calculous cholecystitis with a fistulous connection with colonic specimen.

## 3. Discussion

An extensive review of 160 articles published from 1950 to 2006 by Costi et al. [4] revealed only 231 cases of CCF with a distribution over the different decades increased from 1950 to nowadays.

Despite the fact that CCF often represents a late complication of gallstone disease, it can also occur as a consequence of peptic ulcer disease, Crohn's disease, malignancy, or trauma [4, 5]. The exact aetiology of CCF secondary to gallstone disease is unclear. Glenn et al. [1] proposed that acute inflammation of the gallbladder with obstruction of the cystic duct allows adhesion of the gallbladder to the contiguous organs, most frequently the duodenum. Recurrent acute cholecystitis promotes ulceration and ischaemia of the wall of the gallbladder and the adjacent organs, resulting in further erosion and ultimately fistulation. 

Patients with CCF often present with symptoms of cholecystitis, and preoperative diagnostic tools often fail to show the fistula. 

Sometimes, the complications of bilioenteric fistulas as well as ascending cholangitis, gallstone ileus, weight loss, malabsorption syndrome, gastrointestinal bleeding, and malignancy may suggest a diagnosis of CCF. The most common presenting symptoms of nonobstructing biliary-enteric fistulas are abdominal pain, nausea, and diarrhea. Diarrhea and weight loss can be explained due to the fact that a cholecystocolonic fistula can affect the enterohepatic circulation, leading to a malabsorption syndrome and an increase in the secretion of water and electrolytes from the colon. Bile loss can be partially compensated with an increased hepatic bile acid synthesis. But when the loss is greater than what the liver can compensate, dietary fat solubilization is compromised, leading to steatorrhea [5]. A cholecystocolonic fistula can cause a large-bowel obstruction with stone impaction at rectosigmoid diverticula [5]. Preoperative studies may include ultrasound, CT scan, MR, ERCP, and barium enema, but a proper diagnosis is often achieved intraoperatively [2, 3]. Pneumobilia has been considered to be associated with CCF [5] especially if the gallbladder is atrophic and anatomically adjacent to another organ on computed tomography or ultrasound. However, Yamashita et al. [6] reported that ERCP was the most accurate diagnostic modality of CCF. Wang et al. [7] were able to illustrate CCF using ultrasound, ERCP, and magnetic resonance imaging techniques in 50% of cases.

However, preoperative diagnosis of CCF is very difficult and a misdiagnosis may result in a challenging situation for the surgeon, who is forced to switch from an elective cholecystectomy to a complex procedure that usually involves adhesiolysis and colonic resection. 

For these reasons, the gold standard treatment for nonobstructing biliary-enteric fistulas should be an open cholecystectomy with the closure of the fistula.

Some aspects of recently proposed surgical treatments for uncomplicated CCF have been analyzed, namely, the effectiveness of the laparoscopic procedure, the sequence of resections (cholecystectomy and colonic resection), the modality of colonic suturing, and the potential need for a diversion. Since 1994, a very small number of articles [2, 3, 8, 9] have reported a laparoscopic treatment of CCF. Although these authors supported the feasibility of the entire procedure by the laparoscopic approach, some of them reported a long operating time and, despite the small series of patients, a considerable number of conversions due to iatrogenic colonic perforation [2, 3, 9]. Despite a recent trend towards the laparoscopic accomplishment of the procedure for cholecystoenteric fistula, a multicenter study [2] reported a very high rate of early conversion (55%). Indeed, the avulsion of cholecystoenteric fistulas during laparoscopic blunt dissection is not a rare event [3], and its intraoperative management (intracorporeal “manual” suture) may be a demanding skill for average laparoscopic surgeons to perform on a malacic colonic wall. For these reasons, when a CCF is detected incidentally during a routine laparoscopic cholecystectomy, it could be approached with a laparotomy avoiding long operating time and serious intraoperative complications.

Our case was particular because the patient was a young female, the symptoms were quite absent, and there were no previous episodes of acute cholecystitis. Moreover, all the imaging techniques failed to show a CCF. Preoperatively, CCF cannot be suspected. Thus, the disease was approached laparoscopically. Intraoperatively, the suspect of a CCF was due to the tightly adherent transverse colonic loop to cystic duct and gallbladder, and this finding suggested the conversion to laparotomy.

In conclusion, the data reported in the literature allowed to recognize some peculiar aspects of the CCF. In the presence of repeated episodes of cholecystitis especially associated with CBD stones and also in the absence of specific symptoms like diarrhea and without the presence of aerobilia, the suspect of a CCF must be considered. 

In these cases as well as in the cases discovered intraoperatively, the surgeon will find a “surgical dilemma” due to a very complex pathology to treat laparoscopically for which it will almost be necessary to perform a difficult laparotomic cholecystectomy and colonic resection with consequent increase of the operating time and postoperative complications.
